# Evidence-Based Decision Making in Public Health: Capacity Building for Public Health Students at King Saud University in Riyadh

**DOI:** 10.1155/2015/576953

**Published:** 2015-12-08

**Authors:** Hayfaa A. Wahabi, Amna Rehana Siddiqui, Ashry G. Mohamed, Ali M. Al-hazmi, Nasriah Zakaria, Lubna A. Al-Ansary

**Affiliations:** ^1^Chair of Evidence-Based Healthcare and Knowledge Translation, College of Medicine, King Saud University, P.O. Box 2925 (Internal Code 34), Riyadh 11461, Saudi Arabia; ^2^Department of Family and Community Medicine, College of Medicine, King Saud University, P.O. Box 2925 (Internal Code 34), Riyadh 11461, Saudi Arabia; ^3^Prince Sattam Bin Abdul Aziz Research Chair for Epidemiology and Public Health, College of Medicine, King Saud University, P.O. Box 2925 (Internal Code 34), Riyadh 11461, Saudi Arabia; ^4^Medical Informatics & E-Learning Unit, Medical Education Department, College of Medicine, King Saud University, P.O. Box 2925 (Internal Code 34), Riyadh 11461, Saudi Arabia; ^5^Research Chair in Health Informatics and Promotion, College of Medicine, King Saud University, P.O. Box 2925 (Internal Code 34), Riyadh 11461, Saudi Arabia; ^6^School of Computer Sciences, Universiti Sains Malaysia, 11800 Penang, Malaysia

## Abstract

Translation of research evidence into public health programs is lagging in Eastern Mediterranean Region. Graduate level public health curriculum at King Saud University (KSU), College of Medicine, Riyadh, is designed to equip students to integrate best available evidence in public health decision making. The objectives of study were to explore students' opinion about the evidence based public health (EBPH) courses and to survey the knowledge, opinion, and attitude of the students towards EBPH and perceived barriers for implementation of EBPH in decision making in public health. EBPH courses are designed based on a sequential framework. A survey was conducted at the completion of EBPH courses. Forty-five graduate students were invited to complete a validated self-administered questionnaire. It included questions about demography, opinion, and attitude towards EBPH and perceived barriers towards implementation of EBPH in the work environment. The response rate was 73%. Mean age of students was 30.1 (SD 2.3) years, and 51% were males. More than 80% had sound knowledge and could appreciate the importance of EBPH. The main perceived barriers to incorporate EBPH in decision making were lack of system of communication between researchers and policy makers and scarcity of research publications related to the public health problems.

## 1. Introduction

Evidence-based public health (EBPH) as a concept has evolved during less than two decades to a consensus about decision in public health programs and policies to be based on scientific evidence, available resources, and context [1]. Brownson et al. defined EBPH as “the development, implementation, and evaluation of effective programs and policies in public health through application of principles of scientific reasoning including systematic use of data and information systems and appropriate use of program planning models” [2].

EBPH has important role in strengthening the national health system; it supports effective interventions and hence provides the ground for evidence-based distribution of resources and workforce. Recently there is increased interest, worldwide, in evidence-based health policy and translation of research to action [3, 4]. Many models have been created to facilitate translation of evidence to health policy or clinical decision [5]. Most of these models are based on knowledge about prioritizing health service needs, creating or finding and evaluating necessary evidence, and developing strategies for implementation and evaluation [5, 6].

Although many countries in the Eastern Mediterranean Region (EMR) are lagging behind in biomedical research publications [7] and translation of research evidence into health policy and programs [8, 9], the efforts have been limited in building such capacity [10, 11].

One of the main obstacles to the implementation of EBPH is the lack of knowledge and skills needed for translation of evidence into programs and policy [12]. In the Arab world, a few reports were published about workshops designed to build capacity in EBPH and knowledge translation for healthcare professionals, managers, and policy makers [10, 11]. To scale up EBPH movement in the Arab world, efforts should focus on introduction of EBPH into the postgraduate public health curricula in addition to the workshops designed for professionals and policy makers.

The Department of Family and Community Medicine in College of Medicine at King Saud University (KSU) runs a set of two postgraduate programs, namely, Master of Public Health (MPH) and Saudi Board in Community Medicine (SBCM) residency program. The two-year MPH program was established three years ago and the four-year SBCM program was established four years ago. Both postgraduate courses aim to provide high quality public health professionals with sound knowledge on determinants of health in Saudi Arabia and other Arab communities. In addition to the skills and knowledge of clinical and field epidemiology and medical informatics, the postgraduate programs are designed to equip the graduate with sound knowledge in the use of research evidence in decision making. The program includes courses in EBPH and advanced epidemiology to use results of epidemiological studies and trials to provide evidence for decision making. Courses on how to use and conduct systematic reviews are integrated in both programs. The framework developed by O'Neall and Brownson for teaching public healthcare professionals EBPH [13] is used to deliver the curriculum. This paper reports on our experience in teaching EBPH.

## 2. Objectives

The objectives of this paper are as follows:

1. To describe the activities for capacity building in evidence informed public health decision making in the postgraduate curricula of public health in College of Medicine at King Saud University.
2. To investigate the opinion of the students about the courses of evidence-based public health with respect to usefulness of the content for future students' career, timing of the courses modules in the curriculum, and relevance of the assessment to the content.
3. To explore the knowledge, opinion, and attitude of the students towards evidence-based public health and perceived barriers for implementation of EBPH.

## 3. Methods

**Figure 1:**
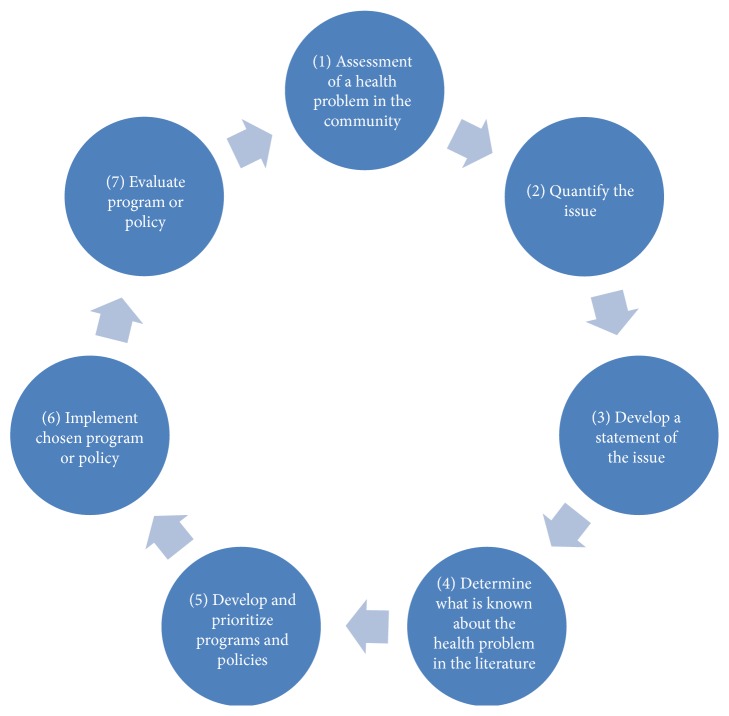
Sequential training framework for evidence-based public health. Adapted from Brownson et al. [1].

The EBPH and systematic review courses are strategically distributed at the end of the first and beginning of second year of the master program and during the third year of the SBCM program, following the basic epidemiology, biostatistics, research methodology, bioinformatics, and health economics courses (Tables 1 and 2). The design of the courses and the methods of instruction are based on the sequential framework developed by O'Neall and Brownson and detailed by Maylahn et al. for teaching EBPH (Figure 1) [13, 14]. The courses provide knowledge and skills in the following domains:

1. Definition of public health issue in the Saudi community.
2. Quantifying the public health problem in local context.
3. Review of the literature and identification of effective interventions.
4. Integrating colloquial knowledge to the decision about program or policy.
5. Developing an intervention program based on evidence.
6. Development of an implementation plan.
7. Evaluation of processes and outcomes.

The method of instruction for these courses follows the principles of adult learning including learning through problem solving, active involvement of students, and integrating the experiences of faculty and students in discussion [15]. Course material was delivered through case scenarios in addition to didactic lectures, open discussion, and hands-on practical sessions. Colloquial knowledge sources are national surveys, reports, registries, or published papers on local population. If such information is not available, students are encouraged to suggest quantitative and/or qualitative studies to fill the knowledge gap.

Evaluation of effectiveness of the courses in building capacity in EBPH was based on the final examination score for the students of a case scenario pertinent to one of the public health problems in their community (the Appendix) and a self-administered questionnaire, based on Likert scale (agree, disagree, or uncertain) about

1. students' opinion and attitude towards EBPH-related courses and the methods of instruction and assessment;
2. students' knowledge, attitude, and opinion towards EBPH;
3. barriers to the implementation of EBPH in the Arab world.

The questionnaire contained three parts; the first part was composed of questions about the demographic characteristics of the student such as age, gender, and years since graduation. The second part included questions about knowledge, attitude, and opinion of the student about EBPH. This part of the questionnaire was developed based on the validated questionnaire developed by De Vito et al. [16]. The third part of the questionnaire was based on the survey developed by El-Jardali et al. on perceived barriers to implementation of evidence-based health policy in the Middle Eastern countries [17]. For both types of graduate student groups the questionnaire was administered two months after completing the EBPH courses.

## 4. Results

Thirty-three students from both programs, who completed the core and the supporting courses (Tables 1 and 2), were included in the evaluation. The response rate was 73%. The demographic characteristics of the students are shown in Table 3. Most of the students have worked before joining the postgraduate program and 36% of them were expected to join the Ministry of Health following graduation (Table 3). More than 70% of the students in the two programs were medical doctors.

The mean score on the case scenario examination, as part of the evaluation of the students' gained skills and knowledge of EBPH, was 88% with 92% as the highest and 78% as the lowest score. Most of the students agreed that the EBPH courses were relevant to their future career and practice (100%), the courses were well situated in the curriculum (91%), and the assessment methods met the course objectives (80%). However great proportion of the students (42.8%) believed that the time allocated for teaching some of the courses such as the systematic review course was not enough and should be extended.

The students' self-evaluation of their knowledge in EBPH is shown in Table 4. About 80–90% of the students agreed that they know most of the steps for EBPH; however only over 60% were confident about their skills of critical appraisal. A great majority (85–94%) know the value of randomized controlled trails, systematic reviews, and observational studies in the process of decision making in public health.

With respect to students' opinion and attitude towards implementation of EBPH in the Arab countries, only 67% believed that EBPH is suitable for implementation in the Arab countries, while 27% were uncertain about such implementation (Table 5). More than 80% of the students agreed that in their new position as public health professionals they would be able to pursue EBPH, using research evidence (80–85%) including systematic reviews (100%).

With respect to students' perceived barriers for implementation of EBPH most of the students agreed that “lack of forum of communication between researchers and policy makers” and “lack of clear system and programs to incorporate evidence into policy” (88–91%) are the main barriers for implementation of EBPH in Arab countries. However, 67% agreed that lack of culture of integrating decision and research evidence is a barrier. Less than 50% of the students agreed that lack of budget for research and lack of well qualified researchers and academicians are barriers to EBPH implementation. Nearly 90% of the students agreed that lack of public health related research in Arab countries constitutes a barrier to EBPH practice (Table 6).

## 5. Discussion

Our results demonstrated that the postgraduate public health students at KSU have positive attitude towards EBPH and towards its curriculum. In addition, it showed that they have sound knowledge about study designs, which can create high level of evidence such as systematic reviews and randomized controlled trials and the use of such evidence in decision making. The survey demonstrated that students, in both programs, are aware of the challenges facing implementation of EBPH in the Arab world.

Similar positive attitude towards EBPH was reported from researchers and policy makers in EMR [17, 18]; however knowledge about evidence-based decision making and knowledge translation was found to be modest among researchers from the same region [8], which supports the importance of our initiative of systematically teaching EBPH to graduate students.

The characteristics of the students in this study establish a solid foundation for capacity building in EBPH in Saudi Arabia and the Arab world. The mix of their basic degrees and their different professional career position as doctors, nurses, health educators, and pharmacists widen the scope of implementation of EBPH in different disciplines in the health system. In addition, the mix creates the suitable climate for dissemination of the culture of EBPH among healthcare professionals and policy makers. The lack of such climate is reported by the participants and others [8], to be a major obstacle for implementing EBPH in programs and policies.

The knowledge and skill mix of the students positively influenced the learning process by creating a multidisciplinary group necessary for the initial steps of developing a statement and quantifying the health issue in our adopted educational framework (Figure 1). In addition, most of the students reported the Ministry of Health as their future employer, which gives them a potential advantage to gear decision making in public health to be evidence-based, from their future career position.

The participants in this study were aware of the challenges facing the implementation of EBPH in the Arab world. They were realistic about the readiness of the Arab world for EBPH, an opinion probably based on their experience as professionals before they joined the postgraduate programs. They listed the lack of an official platform and system of communication between researchers and decision makers for the implementation of EBPH as the main barriers (Table 6). Similar views were expressed by researchers in the East Mediterranean Region [17] and in other developing countries [19]. Although policy makers in EMR considered absence of administrative structure as one reason for lack of EBPH in the Arab countries, they equally acknowledged limited funding, donors' organizations pressure, and delays in reporting the needed evidence as the main barriers to the implementation of EBPH [18]. El-Jardali et al. found complete lack of structural process of using evidence to advise decision in public health in many Arab countries [20]. However, in the same articles they suggested approaches to incorporate evidence into decision making in Arab countries including development of national strategic plan and scaling up of relevant research [20].

Other reported barriers, which could be influenced by our teaching model of public health students, include isolation of researchers and lack of skills for evidence retrieval and production [21]. We believe that developing the trained workforce, during postgraduate educations, who believe in EBPH and who have the needed skills and knowledge to search for evidence and to incorporate it into policy, is a major step in the right direction to overcome many barriers facing the implementation of EBPH in the Arab world.

The framework we adopted for teaching EBPH has proven its effectiveness in building capacity for EBPH among professionals of public health in other countries, evident by the increased use of evidence in decision making supported by increased competencies and a decrease in the knowledge gaps [22]. However, our students frequently face challenges in finding evidence that is pertinent to the population in Saudi Arabia or the region to reach a context sensitive decision. Such challenges are due to lack of surveillance systems and paucity of relevant biomedical literature in the Arab world. Nevertheless, the instructors use these challenges to promote production of relevant research among the students to overcome another barrier for EBPH.

This paper is one of the few reports which describe interventions to address the problem of knowledge translations in public health in the Arab world.

We are aware of the limitations of this study including the small number of participants and the reporting of one centre experience; however we have invited all the students in both programs who completed the core and the EBPH courses to participate and we had a response rate of 73%. Another limitation is the lack of open questions in the survey, which could have given the students the chance to elaborate on their opinion about the EBPH courses and barriers to implementation of EBPH. Additional limitation is the lack of instructors' opinion about teaching EBPH. We acknowledged that the true success of the EBPH courses and framework should be measured by the proportion of evidence-based decision making the graduate students make in the field; however as the programs are just starting such study is planned in the future.

## 6. Conclusion

The postgraduate programs in public health and community medicine at King Saud University, College of Medicine, Riyadh, participate in capacity building in evidence-based decision making in public health. The graduate level students in public health have a positive attitude towards EBPH and they are aware of the obstacles facing its implementation.

## Figures and Tables

**Table 1:** Core courses for evidence-based public health MPH/SBCM curricula.

Course	Description	Competences learned
Basic evidence-based healthcare	The course describes the basic concepts of evidence-based decision making, type of evidence, and sources for evidence in public health	(i) Searching the main biomedical literature databases (ii) Basic evaluation of evidence according to study design and relevance to public health problems

Advanced evidence-based healthcare	The course introduces the students to critical appraisal of the main study designs; in addition it introduces systematic reviews, practice guidelines, and policy briefs	Evaluating and summarizing the evidence

Systematic review	The course is in depth teaching on how to use evidence from systematic reviews for decision making. It includes quantifying the public health problem by using local data, formulating answerable question for effective intervention, searching for relevant systematic reviews, critically appraising the systematic review, and deciding on the impact of the intervention and if that impact is expected to address the public health problem.	(i) Searching and evaluating relevant systematic review for intervention (ii) Assessing the applicability of the intervention to address the local health problem

**Table 2:** Supporting courses for evidence-based public health in the MPH/SBCM curricula.

Course	Description of relevant contents	Competences learned
Research methodology	The course describes different types of quantitative and qualitative study designs	Characteristics, applications, and limitations of different study design and their uses as evidence as epidemiological tools to detect and quantify public health problems

Health economics	Principles of health economics	(i) Economic evaluation of intervention (ii) Choosing between interventions (iii) Program evaluation

Health policy	Principles of health policy and programs development	Developing and evaluating programs for public health

Health informatics	Surveillance system and registries	Use of local data for quantifying public health problems, monitoring, and evaluation of programs for intervention

**Table 3:** Characteristics of the MPH/SBCM students.

Character	Number (%)	Mean ± SD	Range
Age (years)		30.1 ± 2.3	25–37
Gender (male)	17 (51%)		
University degree			
Medical doctor	24 (73%)		
Nurse/health educator	5 (15%)		
Pharmacist	2 (6%)		
Other	2 (6%)		
Sponsoring body for the MPH/SBCM			
Ministry of Health	23 (70%)		
University	7 (21%)		
Others	3 (9%)		
Years since graduation from university		5.3 ± 4.5	2–13
Years of working before joining the MPH/SBCM		5.0 ± 3.2	0–10
Type of work before joining MPH			
Clinical staff	17 (52%)		
University staff	3 (9%)		
Ministry of Health staff	10 (30%)		
Not worked before	3 (9%)		
Expected future position			
Clinical staff	1 (3%)		
University staff	6 (18%)		
Ministry of Health staff	12 (36%)		
Uncertain	13 (40%)		
Other	1 (3%)		

**Table 4:** Students self-evaluated knowledge and skills after completion of EBPH courses.

Skill or knowledge	Agree	Disagree	Uncertain
*After completing the EBPH courses*			
I can formulate a searchable public health problem	30 (91%)	—	3 (9%)
Search the relevant databases for the highest available evidence	29 (88%)	1 (3%)	3 (9%)
Appraise the different types of studies and trials for external and internal validity	21 (64%)	4 (12%)	8 (24%)
Evaluate the impact of intervention by interpreting the relative risk and odds ratio	26 (79%)	1 (3%)	6 (18%)

Public health interventions require effective evaluations of health interventions carried out through research evidence	31 (94%)	—	2 (6%)

Randomized controlled trials and systematic reviews are tools to demonstrate the efficacy of public preventive and curative health interventions	30 (91%)	—	3 (9%)

Observational studies and surveillance data are credible source of evidence in EBPH	28 (85%)	2 (6%)	3 (9%)

Relative risk and odds ratio are measures used to quantify the effect of health interventions	31 (94%)	—	2 (6%)

Meta-analysis combines the results of different individual studies with the purpose of integrating the findings	31 (94%)	1 (3%)	1 (3%)

EBPH: evidence-based public health.

**Table 5:** Opinion and attitude of students towards evidence-based public health.

Statement	Agree	Disagree	Uncertain
EBPH is not suitable for application in the Arab countries	2 (6%)	22 (67%)	9 (27%)
In my new position as a public health officer I will be able to make decision for intervention based on evidence	27 (82%)	2 (6%)	4 (12%)
Systematic reviews contribute significantly to knowledge about prevention and treatment of disease	33 (100%)	—	—
Application of research evidence in public health improves the health status of community	28 (85%)	1 (3%)	4 (12%)
Public health interventions require effective evaluation of health interventions carried out through research evidence	29 (88%)	—	4 (12%)
Decisions in public health cannot be based on the results of randomized controlled trials and meta-analysis but rather on the available budget	10 (30%)	13 (40%)	10 (30%)
Systematic reviews are useful tool for decision making in public health	30 (91%)	—	3 (9%)

EBPH: evidence-based public health.

**Table 6:** Perceived barriers to evidence-based public health implementation.

Barrier	Agree	Disagree	Uncertain
Lack of forum of communication between researchers and public health decision makers	29 (88%)	—	4 (12%)
Lack of clear system and programs to incorporate evidence into decision	30 (91%)	1 (3%)	2 (6%)
Lack of well qualified researchers and academicians in my working position	10 (30%)	10 (30%)	13 (40%)
Lack of budget for research	14 (42%)	8 (24%)	11 (34%)
Lack of culture of integrating research evidence into programs	22 (67%)	6 (18%)	5 (15%)
Lack of public health related research publication in the Arab world	29 (88%)	2 (6%)	2 (6%)

## Appendix

### Case Scenario

It is estimated that by 2020, road traffic crashes (RTC) will have moved from ninth to third in the world ranking of burden of disease, as measured in disability adjusted life years. The prevention of road traffic injuries is of global public health importance. Measures aimed at reducing traffic speed are considered essential to preventing road injuries; the use of speed cameras is one such measure.

The chairperson of the committee for prevention of RTC in the Saudi Ministry of Health, asked you to assess whether the use of speed cameras reduces the incidence of speeding, road traffic crashes, injuries and deaths.

Please answer the following questions:

1. Where do you look for information about the burden of RTC in Saudi Arabia?
2. Which measure will you be using to quantify the burden of RTC in Saudi Arabia
3. Formulate* PICO* (population, intervention, comparison, and outcome) for a search for a systematic review, which examines the effectiveness of the proposed intervention for prevention of RTC?
4. List 3 databases you are going to search to find evidence, which address the search question
5. Your search in Pubmed resulted in more than 9000 citations, how are you going to reduce this number of citations?

By the end of your search, you selected a systematic review, which addresses your search question, from the Cochrane Library entitled (*Speed cameras for the prevention of road traffic injuries and deaths*), Cochrane Database Syst Rev. 2010 Nov 10;(11):CD004607.

You decided to critically appraise this systematic review.

1. List three important items in the methodology of the systematic review, which you are going to look for, to examine the validity of the review.

The results of this systematic review are (Thirty-five studies met the inclusion criteria. Compared with controls, the relative reduction in average speed ranged from 1% to 15% and the reduction in proportion of vehicles speeding ranged from 14% to 65%. Near camera sites, the pre/post reductions ranged from 8% to 49% for all crashes, and 44% to 11% for fatal and serious injury crashes. Compared with controls, the relative improvement in pre/post injury crash proportions ranged from 8% to 50%)

1. From the pooled data what is the reduction in the proportion of vehicle speeding?
2. From the pooled data what is the reduction in the proportion of fatal and serious injuries crashes?
3. If the evaluation of the evidence is moderate to strong and considering the above effect size, do you think implementation of speed cameras is going to be effective in Saudi Arabia given the high prevalence of fatal and serious car crashes in the Kingdom?
4. Outline an implementation plan for the speed Cameras in Riyadh the capital city of Saudi Arabia.
5. Using the logic model outline how you are going to evaluate the process and the outcomes of your implemented program.
